# Intra-operative assessment of toric intra-ocular lens implantation

**DOI:** 10.4103/0301-4738.73726

**Published:** 2011

**Authors:** Ioannis T Tsinopoulos, Chrysanthos Symeonidis, Konstantinos T Tsaousis, Dimitris Tsakpinis, Nikolaos G Ziakas, Stavros A Dimitrakos

**Affiliations:** 2^nd^ Department of Ophthalmology, Medical School, Aristotle University of Thessaloniki, Thessaloniki, Greece

**Keywords:** Toric intraocular lens, toric lens implantation assessment procedure, surgical technique

## Abstract

We report a new procedure for intra-operative toric intra-ocular lens (IOL) axis assessment in order to achieve optimal implantation. IOL implantation procedure was directly recorded. An assessor estimated the angle formed by the marked 0–180 axis and the toric IOL axis after implantation with the use of the appropriate software. If IOL implantation was assessed to be inaccurate, the surgeon was advised to correct IOL positioning by rotating the IOL clockwise. The assessment procedure was repeated until accurate IOL positioning was achieved.

Intra-operative correction of corneal astigmatism was developed in the 1990s[1] but was considerably improved in the beginning of the past decade.[2] In order to achieve the desired outcome, accurate pre-operative data (axial length, keratometric data and accurate intraocular lens (IOL) power calculation with the use of the appropriate biometric formula) and proper surgical technique are required. All existing techniques are based on the fact that the IOL is implanted in the desired axis, as marked pre-operatively.

The growing use of the toric IOL in everyday clinical practice revealed the need for an accurate intra-operative IOL assessment procedure.[3] The aim of this paper is to present an intra-operative assessment method/procedure of accurate toric IOL implantation.

## Materials and Methods

The patient’s keratometric and topographic data were determined with the use of the Magellan Mapper (Nidek, Vigonza, Italy). Corneal topographic data were then inserted in the IOL manufacturer’s software in order to obtain the axis of the desired astigmatic correction as well as the model and refractive power of the IOL. Afterwards, the 0–180 and the desired astigmatic axis correction were marked on the limbus with the use of the appropriate eye marker (Nujits Pre-Op toric reference marker with bubble-AE-2791TBL, ASICO LLC, Westmont, IL, USA) according to the manufacturer’s instructions, with the patient sitting right before the beginning of the surgical procedure. The bubble ensured proper marking.

The surgical procedure was directly streamed and recorded in a computer in the anterior segment unit of our department. “Streamed pictures” is an alternative expression to describe the captured video frames. They provided (due to the availability of different time units and depending on the frame rate of the video recording) optimal conditions, such as angle of viewing and perpendicularity of IOL alignment, in order to calculate the IOL axis position and/or rotation. After sideport incisions and before the use of the viscoelastic, the desired astigmatic axis was marked in the limbus with the use of an Intra-Op toric axis marker, utilizing the acquired keratometric and topographic data and with the marked 0–180 axis as reference (AE-2792, ASICO LLC, Westmont, IL, USA).

**Figure 1:**
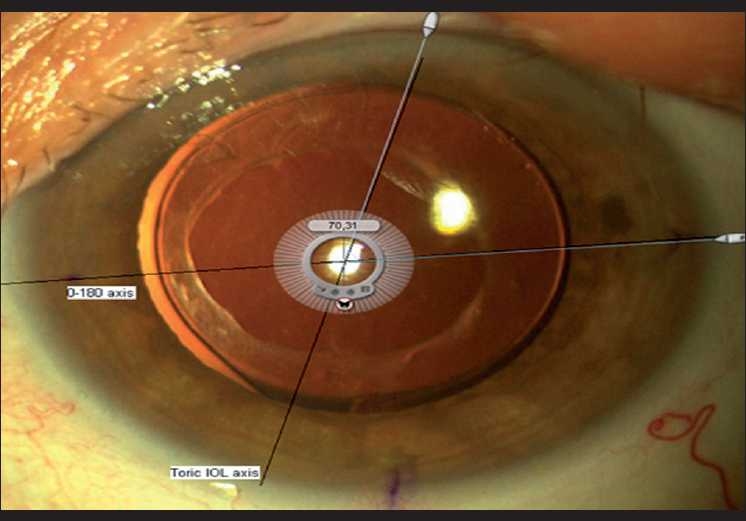
Intra-operative toric implantation assessment with the use of the appropriate software

After an uneventful IOL implantation, the viscoelastic was aspirated thoroughly and an air bubble was placed above the IOL (in order to maintain the IOL centered). The air bubble was then removed with the use of Balanced Salt Solution (BSS) infusion through the sideport. If, at this point, IOL implantation was considered by the surgeon to be perfect, a digital photograph was taken with a digital video camera and evaluated with the use of the following procedure. Medical personnel were responsible for the intra-operative toric IOL assessment. In the digital photograph, a protractor was superimposed on the IOL optic; its positioning was determined by the pre-operatively marked 0–180 axis on the eye and on the toric IOL axis (determined by three marks on each side-Screen Protractor, Iconico, v.4.0, Fig. 1). The assessor then evaluated the angle formed by the two above-mentioned axes in order to verify proper IOL implantation. The duration of this additional procedure was <30 s. The desired correction was not known to the assessor. Thus, bias was reduced. Immediate IOL axis evaluation was quite inconvenient to be performed by the surgeon because he/she operated under sterile conditions and could not operate a personal computer.

If IOL implantation was assessed to be inaccurate, the surgeon was advised to correct IOL positioning by rotating the IOL clockwise. The assessment procedure was repeated until accurate IOL positioning was achieved. It was essential to visualize the 0 and 180-degree mark and the reflection of the microscope lamp on the same corneal plane as well as to use streamed pictures of the surgical procedure and not just intra-operative digital photographs in order to achieve a perfect alignment of the above marks.

In a small case series (21 eyes), in one (5%) toric IOL (Alcon SN60T3) implantation there was an intra-operative deviation of 5 degrees recorded and corrected according to the procedure described above.

## Discussion

Potential factors that may lead to inaccurate toric IOL implantation are incorrect marking of the 0–180 axis and relatively thick marking lines. Furthermore, IOL rotation is still possible following implantation.

A potential merit of our method may be the use of one instrument compared with two (Nuijts Toric axis marker, AE-2740 and Mendez Degree Gauge AE-2765) in the method proposed by Bauer *et al*. Furthermore, our proposed method may serve as an additional precaution measure for an accurate implantation assessment. A possible shortcoming of our assessment method may be the limitations of our instruments; the marker used employs a 10-degree step while the marker used by Bauer *et al*. employs a 5-degree step.[3] Moreover, the axis marker employed by our group does not permit easy intra-operative visualization and assessment of the final IOL axis. These limitations led to the development of an alternative assessment procedure.

The proposed assessment procedure may prolong IOL implantation by only a few minutes and may contribute to increased implantation accuracy in addition to intra-operative direct surgeon visualization because a standardized assessment method is employed. A potential shortcoming of this proposed procedure may be the cost of the video equipment required in order to obtain the streamed pictures. It is well established that every degree of misalignment results in a 3% reduction of astigmatic power in the IOL plane.[2,3] Thus, the magnitude of astigmatic error potentially introduced is dependent on cylinder power itself; the higher the cylinder power, the more accurate one needs to be. According to our experience (21 eyes) and with the use of the above-mentioned marker, the proposed procedure may serve as an additional precaution measure in order to avoid misalignments (especially in the case of higher cylinder powers) and may sufficiently assist in accurate toric IOL implantation as well as positioning assessment.
